# Examining the roles of working memory and visual attention in multiple object tracking expertise

**DOI:** 10.1007/s10339-020-00954-y

**Published:** 2020-02-03

**Authors:** David J. Harris, Mark R. Wilson, Emily M. Crowe, Samuel J. Vine

**Affiliations:** 1 School of Sport and Health Sciences, University of Exeter, St Luke’s Campus, Exeter, EX1 2LU UK; 2 Department of Human Movement Sciences, Vrije Universiteit Amsterdam, Amsterdam Movement Sciences, Amsterdam, The Netherlands

**Keywords:** MOT, Perceptual–cognitive expertise, Eye tracking, Gaze, Sport

## Abstract

When tracking multiple moving targets among visually similar distractors, human observers are capable of distributing attention over several spatial locations. It is unclear, however, whether capacity limitations or perceptual–cognitive abilities are responsible for the development of expertise in multiple object tracking. Across two experiments, we examined the role of working memory and visual attention in tracking expertise. In Experiment 1, individuals who regularly engaged in object tracking sports (soccer and rugby) displayed improved tracking performance, relative to non-tracking sports (swimming, rowing, running) (*p *= 0.02, *η*_*p*_^2^ = 0.163), but no differences in gaze strategy (*ps *> 0.31). In Experiment 2, participants trained on an adaptive object tracking task showed improved tracking performance (*p *= 0.005, *d *= 0.817), but no changes in gaze strategy (*ps *> 0.07). They did, however, show significant improvement in a working memory transfer task (*p *< 0.001, *d *= 0.970). These findings indicate that the development of tracking expertise is more closely linked to processing capacity limits than perceptual–cognitive strategies.

## Introduction

During most daily activities, attention is sequentially allocated and directed towards the most relevant information to execute the current task (Egeth and Yantis 1997; Land 2006). It is also possible, however, to track multiple objects moving simultaneously. The attentional limits of this ability have been extensively studied using the multiple object tracking (MOT) paradigm (Pylyshyn and Storm 1988) where participants track multiple moving targets among visually similar distractors. At the end of a given trial, participants are asked about the status (i.e. target or distractor) of either a single object (probe-one) or all objects (mark-all: Hulleman 2005). Tracking capacity is generally limited to around 4/5 objects (Pylyshyn and Storm 1988), but performance is also dependent on object speed (Verstraten et al. 2000) and proximity (Intriligator and Cavanagh 2001).

Object tracking ability is proposed as an important skill in various dynamic tasks, like driving, and may be particularly relevant to expertise in team sports (Faubert 2013; Mangine et al. 2014; Meyerhoff et al. 2017). There is, however, limited understanding of how expertise in MOT develops. While MOT is highly constrained by capacity limits on visual attention and working memory (WM: Fougnie and Marois 2006; Oksama and Hyönä 2004), expertise in real-world visual attention tasks, such as sport, appears to be driven by perceptual–cognitive abilities,^1^ like gaze behaviour (Mann et al. 2007; Memmert 2009; Memmert et al. 2009). Here, we aim to investigate the relative contributions of WM capacity and gaze strategies to MOT expertise.

In 1988, Pylyshyn and Storm convincingly demonstrated the human ability to keep track of 4 to 5 identical moving targets over several seconds, based only on their spatio-temporal information. MOT is thought to require selective attention to initially identify the items to be tracked, and sustained attention to maintain a representation of each object as it moves among distractors (Drew and Vogel 2008; Pylyshyn and Annan 2006). Importantly for current purposes, tracking is achieved at speeds above which sequential visual fixations can be made (Yantis 1992); hence, if objects are processed in a serial fashion, it is through covert attention switching (Posner et al. 1987)—that is, decoupling the locus of attention from the foveal parts of the visual field. A splitting of attention across multiple targets, sometimes unequally, requires the use of covert attention (Cavanagh and Alvarez 2005; Crowe et al. 2019; Doran et al. 2009), and a significant portion of tracking occurs using peripheral vision (Vater et al. 2017). Consequently, covert attention plays a major role in tracking, but, previous work has also suggested an important role for *overt* visual attention (Fehd and Seiffert 2010; Zelinsky and Neider 2008), which may also be a determining factor in MOT performance.

Despite the commonly reported limit of 4/5 items when tracking (Pylyshyn and Storm 1988; but see Alvarez and Franconeri 2007), there appears to be a high degree of inter-individual variability in this tracking limit, which may link to MOT expertise. Oksama and Hyönä (2004) found tracking limits varied between 2 and 6 items across participants, but with a uniform distribution, suggesting substantial individual differences. Further, individual differences in visual short-term memory and attention switching were found to significantly predict tracking performance. Similarly, individual differences in event-related potentials (ERPs) indicative of sustained and selective attention also predict tracking performance (Drew and Vogel 2008). These findings point to basic attentional differences as the primary determinant of inter-individual variability in tracking performance.

Contrastingly, experience with real-world object tracking may enhance MOT ability. For instance, better tracking ability has been observed in professional radar operators (Allen et al. 2004) and video game players (Green and Bavelier 2006; Trick et al. 2005). Findings from the sporting domain, however, are more mixed. While Mangine et al. (2014) demonstrated a positive relationship between MOT ability and basketball performance, a direct comparison of team sport athletes, non-team athletes and novice athletes found no performance differences in an MOT task (Memmert et al. 2009). Therefore, it remains uncertain whether experience with demanding, real-world tracking tasks is associated with better MOT performance. There are several possible explanations for how expertise in MOT may develop; here, we focus on the role of overt visual attention and WM capacity. As WM plays an executive control role, as well as a storage one (Engle 2002), gaze control is partly dependent on WM. However, we address these as separate abilities to investigate whether expertise is more closely related to covert processing in WM or overt allocation of visual attention.

### Gaze strategies indicative of overt attention allocation

It is generally accepted that there is a large attentional component to MOT (Scholl et al. 2001), much of which is covert (Doran et al. 2009). The role of *overt* attention is, however, less well understood, but can be examined using eye tracking, since shifts of attention and eye movements are closely related (e.g. Findlay and Gilchrist 2003). Initial eye tracking studies have indicated that overt visual attention may indeed play a role in MOT performance (Fehd and Seiffert 2010, 2008; Zelinsky and Neider 2008), with 2 primary visual strategies being identified (Zelinsky and Neider 2008): a *target switching strategy* where participants continually move their point of gaze between targets and a *centroid strategy* where participants keep their gaze on the medial spatial position of the targets.

Centroid looking may facilitate the use of a beneficial perceptual grouping strategy (Yantis 1992). Zelinsky and Neider (2008) demonstrated that when tracking 2 targets, a centroid strategy was predominantly used, but when progressing to 3 and 4 targets, increased time was spent fixating the target objects, indicating a switching strategy. Curiously, however, *within* each condition increased gaze on targets showed a negative relationship with performance. Zelinsky and Neider suggest that switches might occur when a target is in danger of being lost. However, when switching becomes more difficult with increasing target speeds, greater use of the centroid has been found (Huff et al. 2010). Use of the centroid also seems to be important when tracking across changes in viewpoint (Huff et al. 2010). Experimental instructions to adopt a centre looking/grouping strategy have further supported the beneficial effects of centroid looking (Fehd and Seiffert 2010; Yantis 1992). As efficient, goal-directed visual strategies are a characteristic of expertise in a range of real-world tasks (Wilson et al. 2010; Wilson et al. 2015) and are learned with extended practice (Moore et al. 2012), the development of a centroid looking strategy is a viable candidate to explain the acquisition of object tracking expertise.

### Working memory capacity

WM is a temporary, limited capacity store for holding and manipulating information (Baddeley 1986), which means a finite number of ‘perceptual objects’ can be encoded at any one time. Consequently, WM is likely to be a limiting factor for object tracking. Accordingly, the common limit of 4/5 objects found in MOT tasks mirrors the 4-item capacity of visual short-term memory (Cowan 2001; Delvenne and Bruyer 2004). As individual differences in WM capacity exist (Kane and Engle 2002), and it may be trainable (Morrison and Chein 2011), WM is also a candidate for underpinning MOT expertise.

Research has supported an effortful investment of attention during MOT (Cavanagh et al. 2014), indicating reliance on a central resource, such as WM. Additionally, dual-task studies indicate that increasing MOT demands disrupt a concurrent task, and vice versa (Allen et al. 2006; Kunar et al. 2008; Tombu and Seiffert 2008). In particular, this is true for concurrent spatial WM tasks (Zhang et al. 2010). There is evidence from ERP research that the resource underlying WM and tracking ability are similar or related; maintaining object information in WM requires sustained attention to the location of the remembered item (Awh et al. 2000). Further, Oksama and Hyönä (2004) found individual differences in visuospatial short-term and working memory to predict MOT performance. The exact role of WM during MOT is somewhat unclear as Fougnie and Marois (2006) found the decrement caused to a WM task by a 1 target increase in the concurrent MOT task was only 0.5 items, suggesting the existence of an overlap but that MOT was not entirely reliant on WM. Nonetheless, basic processing capacities such as WM seem to play a major role in MOT performance. It remains to be established, however, if tracking expertise is entirely based on WM capacity.

### The current study

In order to examine the role of gaze strategy and working memory capacity in MOT expertise, two experiments were conducted. While previous work has shown that MOT ability can be trained (Faubert 2013), it is unclear what perceptual or cognitive abilities are responsible for this improvement and what type of abilities is responsible for expertise in MOT. Firstly, we examine whether individuals with greater multiple object tracking experience (those regularly playing team sports) display enhanced MOT abilities and whether any advantage is underpinned by differences in gaze behaviour. Secondly, we examine whether improvement in MOT ability through direct training is dependent on changes in gaze strategy or working memory. Together, the two studies address whether naturally occurring expertise is a result of gaze differences (Experiment 1) and whether expertise that is experimentally induced is due to changes in gaze behaviour or WM capacities (Experiment 2).

## Experiment 1

Sport provides a useful setting for understanding how real-world expertise develops in both cognitive and motor skills, and a major focus within sport psychology has been to identify the cognitive abilities that distinguish expert performers from novices (Williams and Ericsson 2005). Rather than enhanced basic attentional abilities, such as visual memory or selective and sustained attention (Abernethy et al. 1994; Memmert et al. 2009) sporting experts display perceptual–cognitive advantages, such as control of visual attention (Lebeau et al. 2016; Mann et al. 2007), anticipation (Savelsbergh et al. 2005) and prediction (Mann et al. 2013). Perceptual–cognitive skills developed playing team sport, where multiple players must be tracked, could underpin object tracking expertise. While initial evidence suggests that real-world MOT experience is linked to better MOT ability in the case of radar operators (Allen et al. 2004), Memmert et al. (2009) found no difference in MOT performance between elite team athletes and non-team athletes or novices. Consequently, we firstly aim to examine whether any differences in MOT performance exist between individuals who face greater demands on real-world object tracking (those regularly playing team sports, such as rugby or soccer) compared to individuals who face lower tracking demands (those playing non-object tracking sports such as rowing or running). Secondly, we aim to assess whether any performance differences in MOT are due to gaze strategy (a perceptual–cognitive skill), as has been found in many areas of sporting expertise (Mann et al. 2007). It was hypothesized that those playing tracking sports would exhibit better MOT performance and that this would be underpinned by differences in gaze behaviour—in particular increased use of a centroid strategy. Additionally, it was predicted that for more difficult trials (higher target travel speed and increased number of targets), participants would show increased switching, and decreased use of the centroid.

## Methods

### Participants

Thirty-one participants from a student population were recruited (25 males, mean age = 22.3 years, SD = 3.4) based on sample size determination through power analysis (G*Power; Faul et al. 2007). Based on a large effect on object tracking performance in a similar independent group design (*η*_*p*_^2^ = 0.34, Green and Bavelier 2006), at least 14 people per group were required to achieve a power of 0.95 for a between group effect in an *F*-test, given *α *= 0.05. Participants were recruited into two independent groups; group 1 (*high tracking sports*) included participants with > 5-year experience playing a team sport (soccer or rugby) on a regular basis (twice per week). Inclusion in group 2 (*low tracking sports*) was based on no regular engagement with *any* sport that involved tracking opponents. This group all participated in regular sport (twice per week) and consisted primarily of rowers, swimmers and runners. While there may be some tracking required in sports like competitive running, the demands on tracking were deemed to be substantially lower than for team sports. University ethical approval was acquired prior to data collection.

### Design

A mixed design was used with sport (high tracking, low tracking) as a between-subject factor and speed (slow, medium, fast) and number (2, 3, 4) of targets as within-subject variables. Outcome measures were target tracking performance (% correct) and gaze variables (gaze directed to centroid, gaze directed to targets and switches between targets).

### Task and equipment

#### MOT task

The multiple object tracking task was based on that used by Jardine and Seiffert (2011). Stimuli were programmed in MATLAB (v2016a) using the Psychophysics Toolbox (Kleiner et al. 2007), powered by a MacBook Pro, and presented on a 22-inch HP 22vx monitor. Participants were seated with their head in a head rest (40 cm from monitor) to eliminate head movement. During the task, 8 identical white discs (0.9 cm diameter equivalent to 1.3° visual angle) were presented against a black background, with targets highlighted by a temporary red outline. Trials varied in the number of targets (2, 3 or 4) and speed of stimulus movement (approximately 7.4, 9.9 or 12.4°s^−1^), the order of which was fully randomized (for an example video see: osf.io/rqpwc/).

#### Eye tracking

Participants’ eye movements were assessed using SMI ETG 2.0 eye tracking glasses (SensoMotoric Instruments, Boston, MA) that record onto a customized Samsung Galaxy smartphone. The glasses are lightweight (76 g) and record binocular eye movements and the visual scene at 60 Hz, to a spatial resolution of 0.5°. Each recording was calibrated across three markers on the computer screen.

### Procedure

**Fig. 1:**
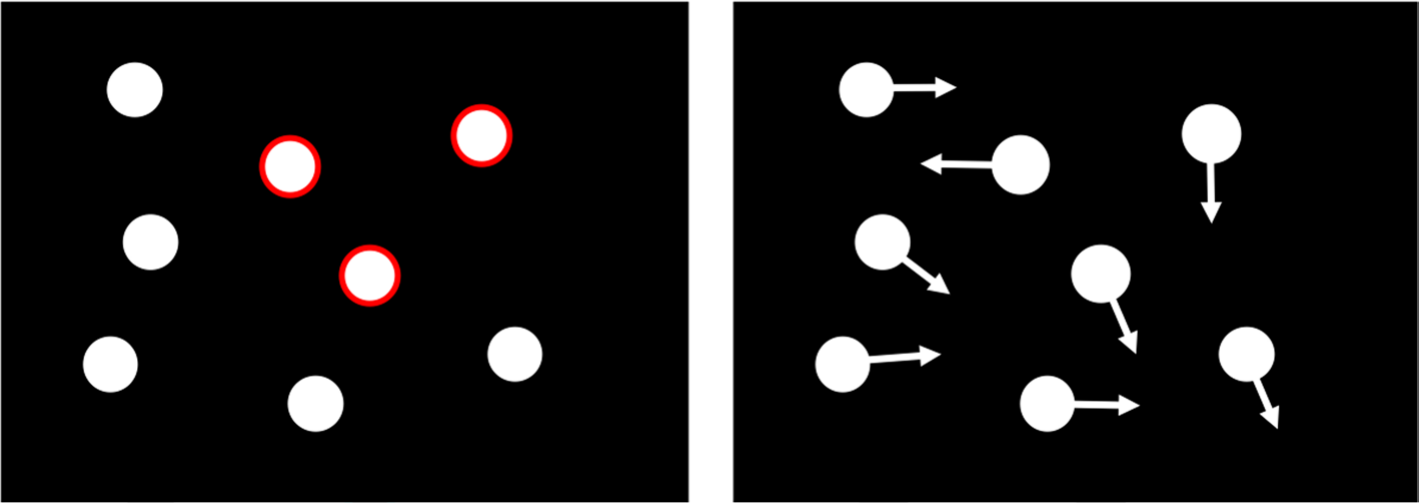
MOT task, showing cue phase (left) and movement phase (right)

Participants were asked to complete 1 practice block (9 trials) and 3 test blocks (9 trials each) of the MOT task with 1-min breaks between each block. Each trial began with a static array of the 8 stimuli randomly placed within an invisible matrix of locations with no overlapping stimuli. Participants were instructed to follow the target discs on all trials, which were simultaneously cued with a surrounding red circle for 2 s (Fig. 1). After the cues disappeared, the display remained static for 1 s and then all objects travelled for 5 s. Items travelled in straight paths, bouncing off the walls of the box and occluded (as opposed to collided) when they converged. Next, participants were required to identify all the targets discs with a mouse click (mark-all method) under no time restriction. Correct targets were indicated after each trial. Accuracy was calculated as the percentage of correctly identified stimuli. The tracking task lasted a maximum of 20 min.

### Data analysis

Gaze data were analysed using MATLAB. Raw data files of gaze coordinates were first obtained through SMI BeGaze 3.7 software (SensoMotoric Instruments, Boston, MA). Coordinates indicating the screen position were identified from marker locations fixated by participants. Coordinate locations of balls in each trial were obtained from MATLAB text files, and a dynamic centroid location (the geometric centre of mass of the target stimuli, as in Fehd and Seiffert 2010) was calculated across each trial. The location of gaze on one of the nine locations (8 balls or centroid) was determined using a k-nearest neighbour procedure (as in Zelinsky and Neider 2008). The percentage of time spent directing gaze to targets, or centroid, and the number of switches between targets was then calculated for each trial. Performance scores were obtained from the MOT program output.

Data analysis was performed in RStudio v1.1.383 (R Core Team 2017). Data were checked for homogeneity of variance (Levene’s test), skewness and kurtosis, and outliers (more than 3 standard deviations from the mean). Performance data substantially deviated from normality and was transformed for analyses using a reflected square root transform. Violations of sphericity were corrected for using a Greenhouse–Geisser correction factor. Bayes factors (BF_10_) were also obtained for main effects and post hoc tests using the *BayesFactor* package (Morey and Rouder 2015) for R. In all analyses, we used the default JZS prior (a Cauchy distribution mean of 0 and a ‘medium’ scale of .5; see Rouder et al. 2012). For consistency, we report *BF*_*10*,_ which corresponds to the amount of evidence in favour of the alternative over the null model. We follow the convention that any *BF*_*10*_ > 3 is evidence for the alternative. Post hoc tests were corrected using the Bonferroni–Holm adjustment. A linear mixed-effects model was run to examine the relationship between gaze strategy and trial success using the lme4 package for (Bates et al. 2014). Successive models were compared using likelihood ratio tests. Gaze analysis scripts and raw data are available from osf.io/rqpwc/.

## Results

### Performance

**Fig. 2:**
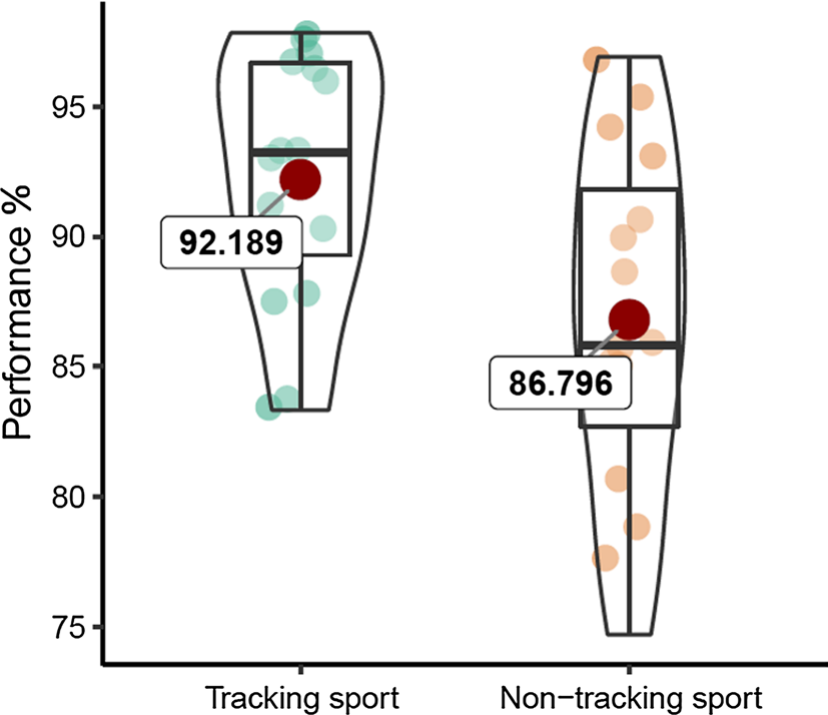
Box plot (median and interquartile range) with individual and mean values (red circle) of overall MOT performance

In order to compare the high tracking group and low tracking group, plus the effect of target number (2, 3 or 4) and target speed (slow, medium, fast), on tracking performance, a 2 (group) × 3 (targets) × 3 (speed) ANOVA was conducted on performance scores (% correct) (Table 1). There was a significant effect of group, *F*(1, 29) = 5.66, *p *= 0.02, *η*_*p*_^2^ = 0.163, BF_10_ = 7.51, with the high tracking sport group showing better performance (Fig. 2). There was a significant effect of target number, *F*(2,58) = 31.04, *p *< 0.001, *η*_*p*_^2^ = 0.517, BF_10_ = 3.73 × 10^6^, with Bonferroni–Holm post hoc tests indicating a decrease in performance from 2 to 3 (*p *< 0.001, *d *= 0.752, BF_10_ = 281.60), 3 to 4 (*p *< 0.001, *d *= 0.620, BF_10_ = 58.10) and 2 to 4 targets (*p *< 0.001, *d *= 1.425, BF_10_ = 2.91 × 10^5^). There was a significant effect of speed, *F*(1.64,47.53) = 9.79, *p *< 0.001, *η*_*p*_^2^ = 0.252, BF_10_ = 154.00, with post hoc tests indicating a decrease in performance from slow to medium (*p *= 0.002, *d *= 0.613, BF_10_ = 91.15) and slow to fast (*p *< 0.001, *d *= 0.846, BF_10_ = 270.80), but not from medium to fast (*p *= 0.34, *d *= 0.174, BF_10_ = 0.27). There were no significant two- or three-way interactions (*p*s > 0.15).

**Table 1:** Mean (and standard deviation) of percentage correct performance in target tracking

Targets	Speed	Team sports	Non-team sports
2	Slow	98.96 (4.17)	94.44 (13.61)
	Medium	95.83 (7.45)	94.44 (8.14)
	Fast	94.79 (10.03)	87.78 (13.31)
3	Slow	96.53 (7.82)	86.12 (18.57)
	Medium	89.59 (12.48)	86.53 (10.60)
	Fast	88.90 (8.11)	89.64 (11.47)
4	Slow	93.23 (6.25)	88.33 (9.86)
	Medium	87.50 (10.97)	77.22 (13.90)
	Fast	84.38 (12.87)	76.67 (13.43)
5	Slow	92.08 (9.50)	90.44 (10.75)
	Medium	87.08 (8.24)	86.11 (9.67)
	Fast	80.42 (11.28)	78.67 (10.45)

### Eye tracking

In order to assess gaze behaviour between groups and across target and speed variations, eye tracking measures were assessed using 2 (group) × 3 (targets) × 3 (speed) ANOVAs.

#### Target looking

**Fig. 3:**
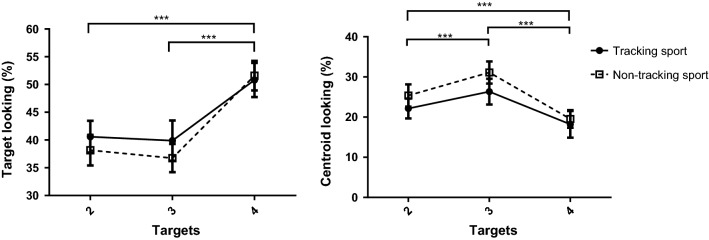
Mean (and standard error) values for the effect of target number on target looking (left) and centroid looking (right). ***p < 0.001

For proportion of time spent directing gaze to target stimuli, there was no effect of group, *F*(1,25) = 0.37, *p *= 0.55, *η*_*p*_^2^ = 0.015, BF_10_ = 0.22. There was also no effect of speed, *F*(2,50) = 0.61, *p *= 0.55, *η*_*p*_^2^ = 0.024, BF_10_ = 0.06. There was a significant effect of target number, *F*(2,51) = 41.08*, p *< 0.001, *η*_*p*_^2^ = 0.611, BF_10_ = 4.15 × 10^10^, with post hoc tests indicating significantly increased time on targets for 4 targets compared to 3 (*p *< 0.001, *d *= 1.605, BF_10_ = 3.79 × 10^5^) or 2 target (*p *< 0.001, *d *= 1.472, BF_10_ = 2.26 × 10^5^) conditions (Fig. 3). There was no difference between 2 and 3 targets (*p *= 0.38, *d *= 0.168, BF_10_ = 0.28). There were no significant two- or three-way interactions (*p*s > 0.16).

#### Centroid looking

For gaze directed to the centroid location, there were no effect of group, *F*(1,25) = 1.10, *p *= 0.31, *η*_*p*_^2^ = 0.042, BF_10_ = 1.01, and no effect of speed, *F*(1.54,38.48) = 1.41, *p *= 0.25, *η*_*p*_^2^ = 0.053, BF_10_ = 0.07. There was an effect of target number*, F*(1.53,38.34) = 15.56, *p *< 0.001, *η*_*p*_^2^ = 0.377, BF_10_ = 1.10 × 10^5^, with post hoc tests showing significantly less gaze directed to the centroid in the 4 target condition than in the 2 (*p *= 0.001, *d *= 0.635, BF_10_ = 2.94) or 3 target conditions (*p *< 0.001, *d *= 1.335, BF_10_ = 559.40) (Fig. 3). There was, however, most gaze directed towards the centroid for 3 targets, significantly more than for 2 targets (*p *< 0.001, *d *= 0.810, BF_10_ = 122.80). There were no significant two- or three-way interactions (*p*s > .31).

#### Target switching

**Fig. 4:**
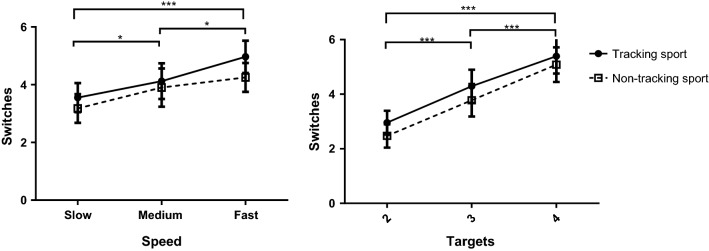
Mean (and standard error) values for the effect of target speed (left) and number (right) on target switching. *p < 0.05 ***p < 0.001

For switches between target stimuli, there was no effect of group, *F*(1,24) = 0.72, *p *= 0.40, *η*_*p*_^2^ = 0.029, BF_10_ = 0.97. There was a significant effect of speed, *F*(2,48) = 8.53, *p *< 0.001, *η*_*p*_^2^ = 0.259, BF_10_ = 8.40, with post hoc tests showing more switches were made at higher speeds, with significant differences between slow and medium (*p *= 0.03, *d *= 0.434, BF_10_ = 1.20), slow and fast (*p *< 0.001, *d *= 1.003, BF_10_ = 182.90) and medium and fast (*p *= 0.03, *d *= 0.485, BF_10_ = 1.50) (Fig. 4). There was also a significant effect of target number, *F*(2,38) = 36.88, *p *< 0.001, *η*_*p*_^2^ = 0.605, BF_10_ = 3.83 × 10^8^), with post hoc tests showing more switches were made for higher target numbers, with significant differences between, 2 and 3 (*p *< 0.001, *d *= 1.027, BF_10_ = 64.09), 2 and 4 (*p *< 0.001, *d *= 1.861, BF_10_ = 2.07 × 10^6^) and 3 and 4 (*p *< 0.001, *d *= 0.932, BF_10_ = 813.81). There were no significant two- or three-way interactions (*p*s > 0.18).

To examine the relationship between gaze strategy and performance (correctly identifying all targets on a trial), a linear mixed-effects model was run (Tables 2 and 3). In the initial model, fixed effects of group, target speed, target number and a target*speed interaction were entered to control for the effect of trial difficulty and sport. The model included by-participant random slopes across levels of target speed and target number, with by-group random effects for slope and intercept across participants (Barr et al. 2013).

**Table 2:** Likelihood ratio tests of fixed effects in the final model

	χ^2^	Df	*p*
Speed	20.29	2	**< 0.001**
Targets	71.25	2	**< 0.001**
Speed × targets	5.55	4	0.23
Group	5.14	1	**0.02**
Centroid looking	6.84	1	**0.009**

Bold values indicate significant effects

**Table 3:** Summary of fixed effects in final model

	*β*	95% CI low	95% CI high	Odds ratio
Intercept	1.35***	0.75	1.95	3.87
Speed—medium†	0.61	− 0.33	1.54	1.84
Speed—slow†	1.70**	0.41	3.00	5.49
2 targets†	− 0.60	− 1.37	0.17	0.55
3 targets†	− 2.00***	− 2.74	− 1.26	0.14
Group	0.54*	0.07	1.01	1.72
Centroid looking	0.26**	0.06	0.45	1.29

†Reference categories were ‘fast’ for speed and ‘4’ for target number

*p < 0.05, **p < 0.01, ***p < 0.001

Full model details are available in supplementary materials

Further to this initial model, the additional effect of eye tracking strategy on task success was examined. The addition of time on targets, *χ*^*2*^(1) = 0.00 *p *= 1.00, or switches, *χ*^*2*^(1) = 0.00, *p *= 1.00, did not improve model fit. Finally, the addition of time directed to the centroid was found to significantly improve the model, *χ*^*2*^(1) = 6.61, *p *= 0.01, indicating centroid looking to be a beneficial strategy, independent of changes in task difficulty.

## Discussion

Experiment 1 compared MOT ability, and gaze strategy, of individuals from high object tracking and low object tracking sports to understand whether differences in gaze underpin the development of expertise in MOT. In line with our primary hypothesis, players of tracking sports (soccer and rugby), showed better object tracking ability, which manifested as a large effect (*η*_p_^2^ = 0.163). This finding is in contrast to that of Memmert et al. (2009), which may be due to Memmert et al. allowing participants to choose a comfortable target speed for the object tracking task. It is possible that better performance among those playing team sports is due to individuals with greater MOT ability being drawn to team sports, but as recent findings indicate MOT to be trainable (Faubert 2013), a development of skill in this area is also highly plausible.

In contrast to our secondary hypothesis that the high tracking sport group would show a more efficient gaze strategy, by focusing on the centroid (Fehd and Seiffert 2010; Zelinsky and Neider 2008), no difference was found in the use of a centroid strategy, or the number of between target switches. This is despite finding that time spent fixating the centroid was a reliable predictor of trial-level success. This lack of difference indicates that the performance advantage shown by the tracking sport group was not dependent on overt visual attention.

Additionally, results were in line with previous findings (Zelinsky and Neider 2008) in showing that participants resort to a target switching strategy, spending more time attending to targets and less time focusing on the centroid, at higher target numbers (Fig. 3). This is despite the overall advantage we found for centroid looking. Also, for increased target numbers and speeds, participants made more switches between targets (Fig. 4), which may be an inefficient strategy, but necessary when targets are in danger of being lost (Zelinsky and Neider 2008).

Overall, these findings suggest that while individuals more experienced with real-world tracking demonstrate enhanced MOT abilities, this is not due to differences in gaze behaviour. Consequently, the underlying ability that accounts for MOT expertise may be a more fundamental cognitive capacity. As WM capacity has been linked to MOT performance (Oksama and Hyönä 2004), it is possible that WM ability could be a feature of MOT expertise. Therefore, study 2 aimed to examine whether improvement in MOT through direct training was related to changes in overt visual attention, or WM capacity.

## Experiment 2

Experiment 1 revealed enhanced MOT performance in individuals participating in sports which place greater demands on object tracking. Similarly, improved MOT performance has been documented in professional radar operators (Allen et al. 2004) and video game players (Green and Bavelier 2006). MOT performance can be improved through direct training (Faubert 2013), but it remains unclear whether this is due to development of processing capacity or through visual attentional strategies. As discussed, previous work has highlighted working memory as a key function, and limiting factor, in MOT (Fougnie and Marois 2006; Oksama and Hyönä 2004). Hence, MOT practice could improve MOT performance though an increase in WM capacity. Indeed, WM improvements following MOT practice have previously been observed (Parsons et al. 2016; Vartanian et al. 2016). In both Experiment 1, and other previous work (Zelinsky and Neider 2008), however, a visual strategy of attending to the centre of mass of target discs (the centroid) was also a predictor of performance. Hence, MOT ability may not only be dependent on the improvement of capacity limitations, like WM, but also overt visual behaviour.

Consequently, Experiment 2 aimed to investigate whether the development of MOT ability through direct training was related to changes in WM and/or gaze strategy. As gaze control is related to expertise in real-world dynamic tasks (Mann et al. 2007; Wilson et al. 2010), and develops with task learning (Moore et al. 2012), it was predicted that gaze strategy would develop with training. In particular, based on the findings of study 1, it was predicted that training would lead to greater use of a centroid looking strategy. Based on the importance of WM capacity in MOT (Oksama and Hyönä 2004) and previous studies showing improvements in WM following MOT training (Parsons et al. 2016; Vartanian et al. 2016), it was predicted that tracking practice would also lead to improvements in WM.

## Methods

### Participants

Thirty-six participants from a student population were recruited (22 females, mean age = 22.5 years, SD = 3.7) based on sample size determination through power analysis (G*Power; Faul et al. 2007). Based on the large effect obtained in Experiment 1 (*η*_*p*_^2^ = 0.163), 15 people per group were required to achieve a power of .95, for independent groups in an *F*-test, given *α* = 0.05. Participants were randomly allocated to training or control groups. University ethical approval was acquired prior to data collection.

### Design

A mixed design was used with training group (adaptive 3D MOT training, control) as a between-subject factor and test (baseline, post) as a within-subject variable. Outcome measures were as in Experiment 1.

### Task and equipment

#### MOT training task

The training task consisted of an adaptive 3D MOT task—known as NeuroTracker (https://neurotracker.net/)—as this particular task has previously been used for training perceptual–cognitive skills in sport (Romeas et al. 2016). Each session consisted of 4 blocks of 20 object tracking trials lasting 10 s each (2-s identification phase, followed by 8 s of movement). The task was presented on a large screen (100 × 150 cm) using a 3D projector (Epson EHTW5650) and active 3D glasses (Epson ELPGS03). Stimuli were 3D yellow balls (approximately 2° visual angle, depending on depth) travelling inside a 76x137 cm cube (covering 48° visual angle). All trials present 4 targets and 4 distractors, with trial speed constantly adapted to provide an optimal level of challenge. If a correct response is given, speed increases, and if an incorrect response is given, speed decreases (see Faubert and Sidebottom, 2012 for more detail). In line with the software guidelines, and due to the adaptive nature of the task, performance was assessed through speed thresholds—the speed at which participants were able to identify all targets correctly 50% of the time.

#### MOT assessment task

Assessment of MOT performance utilized the same task as in Experiment 1. Trials varied in the number of targets (2, 3 or 4) and speed of stimulus movement (approximately 7.4, 9.9 or 12.4°s^−1^), the order of which was fully randomized.

#### Working memory task

At baseline and post-test, participants completed an *n*-back working memory task. The *n*-back task requires participants to decide whether a stimulus in a sequence matches one appearing *n* trials previously. This requires simultaneous storage and manipulation of information and is proposed to measure working memory capacity (Kane and Engle 2002). In task 1, a square moving within a 3 × 3 matrix had to be monitored for 3-back matches, and in task 2, the square had to be monitored for 2-back matches, while auditory stimuli (letters) were simultaneously monitored for 2-back matches (dual *n*-back task). Percentage correct scores were averaged across the two tasks.

### Procedure

On visit 1 (baseline), both groups (training and control) completed the MOT task, with simultaneous eye tracking, from Experiment 1, which consisted of 1 practice block (9 trials) and 2 test blocks (9 trials each) of the MOT task. Participants also completed an *n*-back working memory test (3-back and dual 2-back). The training group then engaged in a 20-min training session (four blocks of 20 trials) on the adaptive 3D MOT task. The training group also returned for four more 20-min training sessions over a period of 12–14 days. The control group did no tracking training during this time (i.e. passive control). Both groups then attended a post-test session, where working memory tests and the MOT task (with eye tracking) were repeated.

### Data analysis

Eye tracking and statistical analysis were performed as in Experiment 1. One single outlying value (more than 3 standard deviations from the mean) was removed from the target switching results. Gaze videos were manually screened for poor calibration or tracking and any that showed poor recordings were removed from the analysis (14% overall drop out). Data were collapsed over target speeds and number.^2^

## Results

### Performance

#### MOT training task

To check for learning on the MOT training task (NeuroTracker), a 2 (test) × 2 (group) mixed ANOVA was run on training speed thresholds. There was found to be a significant effect of test, *F*(1,34) = 54.59, *p *< 0.001, *η*_*p*_^2^ = 0.616, BF_10_ = 28.24, but no effect of training group, *F*(1,34) = 3.30, *p *= 0.08, *η*_*p*_^2^ = 0.088, BF_10_ = 1.58. There was, however, a significant interaction effect, *F*(1,34) = 37.24, *p *< 0.001, *η*_*p*_^2^ = 0.523, BF_10_ = 59.37. Post hoc tests revealed a significant improvement in the training group, (*p *< 0.001, *d *= 1.82, BF_10_ = 2.93 × 10^4^), but not in the control group, (*p *= 0.21, *d *= 0.310, BF_10_ = 0.51).

#### MOT assessment task

**Fig. 5:**
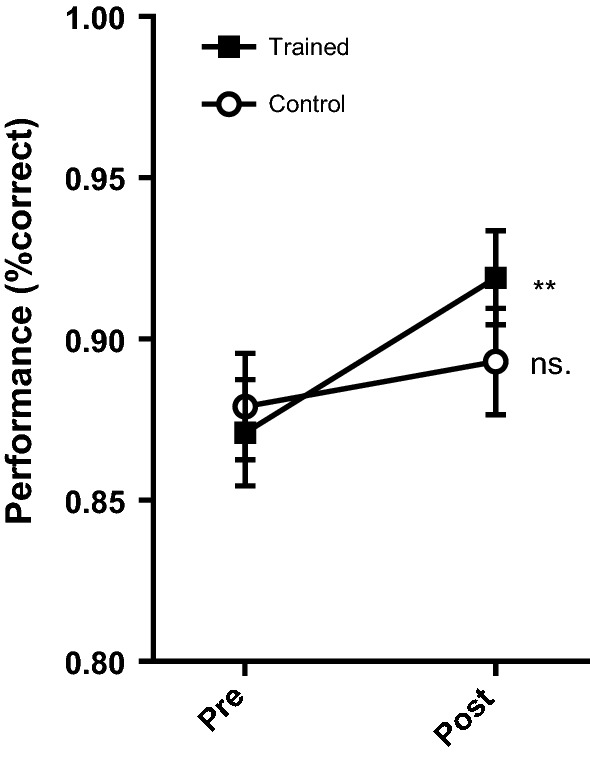
Mean MOT assessment task performance (% correct). Error bars indicate standard error (± 1). ** Change significant at *p* < 0.01; ns. change non-significant

To assess whether adaptive MOT training transferred to improved performance in the MOT assessment task, a 2 (test) × 2 (group) ANOVA was run on transformed performance scores. There were a significant main effect of test *F*(1,32) = 11.28, *p *= 0.002, *η*_*p*_^2^ = 0.261, BF_10_ = 1.35, but no effect of training group, *F*(1,32) = 0.50, *p *= 0.49, *η*_*p*_^2^ = 0.015, BF_10_ = 0.34. There was also a significant interaction effect, *F*(1,32) = 4.81, *p *= 0.04, *η*_*p*_^2^ = 0.131, BF_10_ = 0.53. Post hoc tests revealed a significant improvement in performance among the training group (*p *= 0.005, *d *= 0.817, BF_10_ = 9.47), but not the control group, (*p *= 0.32, *d *= 0.243, BF_10_ = 0.39), indicating that training leads to improved MOT performance (Fig. 5).

### Eye tracking

#### Target looking

To assess the effect of training on gaze behaviour, a 2 (test) × 2 (group) ANOVA was run on time spent fixating target discs. There were no effect of test *F*(1,24) = 0.02, *p *= 0.89, *η*_*p*_^2^ = 0.001, BF_10_ = 0.27, no effect of group, *F*(1,24) = 0.02, *p *= 0.88, *η*_*p*_^2^ = 0.001, BF_10_ = 0.32, and no interaction, *F*(1,24) = 1.96, *p *= 0.17, *η*_*p*_^2^ = 0.075, BF_10_ = 0.08.

#### Centroid looking

A 2 (test) × 2 (group) ANOVA on time spent fixating the centroid location revealed no effect of test *F*(1,24) = 2.12, *p *= 0.16, *η*_*p*_^2^ = 0.081, BF_10_ = 0.59, no effect of group, *F*(1,24) = 2.97, *p *= 0.10, *η*_*p*_^2^ = 0.110, BF_10_ = 0.74, and no interaction, *F*(1,24) = 0.13, *p *= 0.73, *η*_*p*_^2^ = 0.005, BF_10_ = 0.42.

#### Target switching

A 2 (test) × 2 (group) ANOVA on switches of gaze between targets showed no effect of test *F*(1,23) = 0.88, *p *= 0.36, *η*_*p*_^2^ = 0.037, BF_10_ = 0.26, no effect of group, *F*(1,23) = 2.38, *p *= 0.14, *η*_*p*_^2^ = 0.094, BF_10_ = 0.30, and no interaction, *F*(1,23) = 3.76, *p *= 0.07, *η*_*p*_^2^ = 0.141, BF_10_ = 0.09.

### Working memory task

**Fig. 6:**
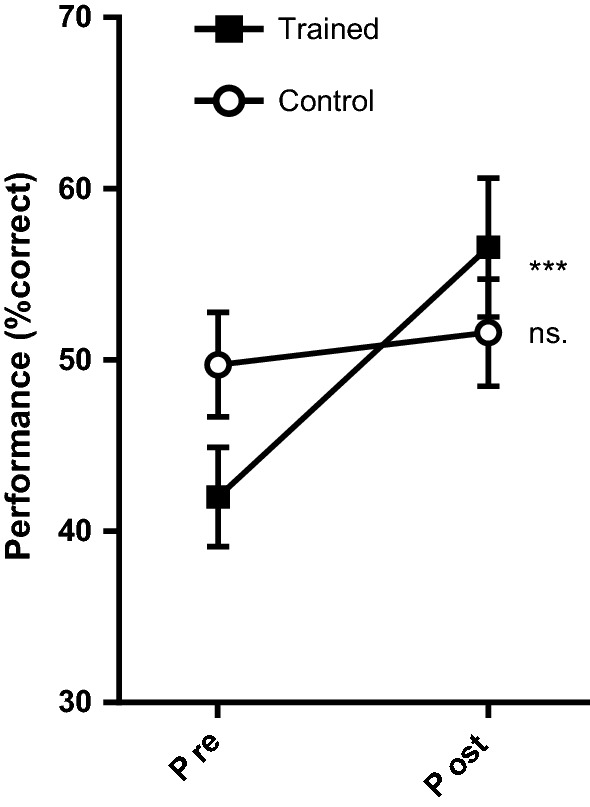
Mean WM task performance (% correct). Error bars indicate standard error (± 1). ***Change significant at *p* < 0.001; ns. change non-significant

A 2 (test) × 2 (group) ANOVA on WM task performance showed a significant main effect of test, *F*(1,34) = 19.44, *p *< 0.001, *η*_*p*_^2^ = 0.364, BF_10_ = 3.64, but no effect of group, *F*(1,34) = 0.08, *p *= 0.79, *η*_*p*_^2^ = 0.002, BF_10_ = 0.26. There was a significant interaction effect, *F*(1,34) = 4.50, *p *= 0.04, *η*_*p*_^2^ = 0.117, BF_10_ = 0.94, with post hoc tests revealing a significant increase in WM performance in the training group (*p *< 0.001, *d *= 0.970, BF_10_ = 49.53), but not in the control group, (*p *= 0.08, *d *= .443, BF_10_ = 1.03)^3^ (Fig. 6).

## Discussion

Experiment 2 investigated whether improvement in MOT ability was related to changes in overt gaze strategy or information processing capacities such as WM. Following training on an adaptive 3D MOT task, a large improvement in MOT performance (*d *= 0.817) was observed in the training group, but not in passive controls. In contrast to our hypothesis, however, there was no change in gaze strategy as a result of training, despite significant improvements in performance. We observed no variation in proportion of gaze directed to the centroid or to target discs, or in the number of switches made between targets. Consequently, the improvements made in tracking performance were not a consequence of gaze behaviour.

Conversely, we observed a significant improvement in performance on the WM task among the training group but not controls. While the Bayes factor for the interaction (0.94) suggested limited evidence for an effect, the effect size was bordering on large (*η*_*p*_^2^ = 0.117) and follow-up tests showed the improvement of the training group to be sizable (*d *= 0.970, BF_10_ = 49.53). This result is in line with previous work which has outlined WM as the central processing resource underpinning tracking (Allen et al. 2006; Tombu and Seiffert 2008) as well as similar improvements in WM following 3D-MOT training (Parsons et al. 2016; Vartanian et al. 2016). Studies examining individual differences in tracking have previously supported attentional and WM capacities as a predictor of MOT performance (Drew and Vogel 2008; Oksama and Hyönä 2004), and the current findings further indicate that the development of MOT expertise may be closely related to WM.

### General discussion

The aim of these studies was to investigate the mechanisms responsible for the development of expertise in multiple object tracking. Much previous work has focused on the attentional requirements of tracking multiple targets (Meyerhoff et al. 2017), but there is limited understanding of whether expertise is dependent on overt visual attention or capacity limitations like WM. Additionally, despite the apparent importance of tracking in dynamic real-world tasks, limited research has linked tracking performance with real-world abilities. Consequently, the current findings have important implications for a theoretical understanding of expert tracking performance as well as applied implications for developing tracking ability.

Our findings across two experiments indicated that while visual strategy was related to trial-by-trial success it was not a feature of expertise in MOT. In Experiment 1, individuals from object tracking sports (i.e. those experienced with real-world MOT) showed better MOT performance, but displayed no differences in gaze strategy. In Experiment 2, training on an adaptive MOT task led to improved MOT performance, but without any changes in gaze strategy, again indicating that expertise may not be dependent on overt visual attention. Experiment 2 also revealed improvements in WM task performance as a result of MOT training, suggesting that improvements in performance were related to corresponding improvements in WM capacity. Consequently, MOT expertise may be more closely related to processing capacity limits and covert attention than gaze behaviour (Allen et al. 2006; Cavanagh and Alvarez 2005; Doran et al. 2009; Oksama and Hyönä 2004). These findings contribute to an understanding of tracking expertise, as while tracking can be trained, improvements may not be due to visual strategies, as is the case for many visuomotor skills (Mann et al. 2007).

The limitations on tracking imposed by WM capacity are already well supported, but these findings suggest that it may also play a role in the development of expertise. The use of dual-task paradigms (Tombu and Seiffert 2008) and manipulations of the temporal and spatial difficulties of target discrimination (Alvarez and Franconeri 2007) have previously shown that MOT performance depends on a central, amodal, processing resource, such as WM. Indeed, previous findings have suggested that the typical WM capacity of 5–7 items may be closely linked to the 4–5-object limit on tracking (Cowan 2001; Delvenne and Bruyer 2004). The current findings (Experiment 2) suggest that as WM may impose a limiting factor on tracking, development of expertise may require expansion of this capacity.

The role of WM in tracking is not straightforward, however, as it may contribute to wider attentional processes, as well as providing a central information store. WM is an amodal resource that plays an important role in attentional control (Kane et al. 2001) and may contribute to the regulation of inhibition, selection and sustained attention, all of which are employed during target tracking (Drew and Vogel 2008; Pylyshyn and Annan 2006). As such, development of WM may also contribute to MOT through improved attentional control. It has also been questioned whether WM tasks are a measure of pure capacity or whether such tests may be more reflective of general controlled attentional functions (Engle et al. 1999). Hence, improvement on the WM test found here may indicate increased WM capacity, but could also reflect an improved attentional control ability, both of which are likely to contribute to MOT performance (Ducrocq et al. 2016).

While both experiments suggested that gaze behaviour was not an important factor in expertise, our results did indicate a centroid looking strategy to be a beneficial strategy, in line with previous work (Fehd and Seiffert 2010; Yantis 1992; Zelinsky and Neider 2008). Paying attention to the centroid, and using peripheral vision to track targets, may be a similar strategy to the ‘visual pivot’ that has been identified in sporting tasks. When facing an opponent in karate (Williams and Elliott 1999) or attempting to save a soccer penalty kick (Piras and Vickers 2011), an effective visual strategy is to attend to a central location (e.g. the hips) and monitor other visual cues peripherally, as opposed to switching between informative areas. This strategy appears to be beneficial in the traditional MOT paradigm, but future work is required to examine whether this strategy is also employed in real-world object tracking. If findings from the traditional MOT paradigm hold for real-world tasks, the importance of centroid looking (or the visual pivot) suggests opportunities for performance enhancement through methods like feedforward eye movement training (see Vine and Wilson 2011).

The absence of any gaze differences between groups (high vs low tracking sports in Experiment 1 and trained vs untrained in Experiment 2) was somewhat surprising, as perceptual–cognitive skills—measured through indices like fixations rate, goal-directed attention and the ‘quiet eye’ (Mann et al. 2007)—play an important role in visuomotor expertise. It may be the case that the training period was insufficient to see measurable changes in visual behaviour. Acute changes in functional gaze behaviour might also require more explicit teaching of eye movements, as has been found in children with coordination disorders (Miles et al. 2015), surgery (Vine et al. 2012) and aiming sports (Vine and Wilson 2011). Nonetheless, if overt visual attention was driving performance, we would have expected the performance differences, found in both experiments, to be accompanied by changes in gaze.

### Limitations

One limitation to consider when interpreting our findings is the representativeness of the object tracking task for real-world tracking environments. Since a team sport requires an individual to track the position, identity and changing features of an object, the MOT task used in many studies is atypical of the sporting environment. Using modified MOT tasks, Crowe and Kent (*in progress*) revealed that employing a novel index of tracking accuracy—namely participants’ reaction time to respond to a critical event—produced different capacity limits on tracking. This demonstrates the need to develop tracking and training tasks that reflect the real world more closely and, therefore, incorporate aspects such as identity (e.g. team mate, opponent) and features (e.g. body posture indicates a player is about to pass the ball) of targets. For instance, feature-based grouping (such as shirt colour) has been found to occur automatically and to facilitate tracking in target grouping or disrupt tracking through binding targets with distractors (Erlikhman et al. 2013).

When interpreting these findings, it is also important to note that WM capacity of the two groups was not well matched at baseline in Experiment 2, although the difference was non-significant (*p *= 0.21). While the size of the improvement observed in the trained group (*d *= 0.970) suggests that the effect is unlikely to be a result of a regression to the mean, this may have accounted for a portion of the improvement, given the lower baseline. Additionally, Experiment 2 might have benefitted from the addition of an active control group, in case the differences in contact could have influenced performance.

## Conclusions

This study examined expertise in MOT through naturally occurring (object tracking versus non-object tracking sports) and experimentally induced (via MOT training) differences in object tracking ability. Both experiments indicated that while gaze strategies, such as use of the centroid, may be related to trial success, they are not a notable feature of expertise. Changes in working memory, however, were related to improvement in tracking performance, suggesting that fundamental processing capacities may underpin expertise in MOT, rather than overt visual attention.

## Footnotes

1 Perceptual–cognitive skills refer to the ability of an individual to identify, locate and process information in order to select and execute appropriate responses (Marteniuk 1976).

2 As no hypotheses were made regarding target speed and number, and for the sake of brevity, these factors were not included in the analyses. For completeness, analyses with these additional factors are reported in supplementary materials (see osf.io/rqpwc/). There were no interactions of target number or speed with training or group (*p*s > .06).

3 While groups were not well matched at baseline (Fig. 6), this difference was non-significant (*p *= .21). Nonetheless, this dissimilarity could have exaggerated training effects.
